# Information System for Tablets Identification (ISTI)

**DOI:** 10.1080/07853890.2021.1897428

**Published:** 2021-09-28

**Authors:** M. E. Torres, A. Figueiredo, C. Família, I. M. Costa, D. P. Tavares, A. Quintas

**Affiliations:** a Centro de Investigação Interdisciplinar Egas Moniz (CiiEM), Egas Moniz Cooperativa de Ensino Superior, Monte de Caparica, Caparica, Portugal; b Laboratório de Ciências Forenses e Psicológicas Egas Moniz, Centro de Investigação Interdisciplinar Egas Moniz (CiiEM), Egas Moniz Cooperativa de Ensino Superior, Caparica, Portugal; c PharmSci Lab – Innovative Solutions in Pharmaceutical Sciences, Centro de Investigação Interdisciplinar Egas Moniz (CiiEM), Egas Moniz Cooperativa de Ensino Superior, Caparica (CiiEM), Caparica, Portugal

## Abstract

**Introduction:**

This study aims to develop a software application (App) for the identification of drugs of abuse from visual information retrieved from pills: Information System for Tablets Identification (ISTI). The consumption of anxiolytics and hypnotics, in particular, benzodiazepines (BZD) and similar products, as reached record levels in Portugal, with 10.5 million packages of these products being sold in 2018 [1]. The high prescription of BZD presents a risk to public health [2,3]. An Information system like ISTI will provide an enormous importance on pills’ identification at crime scenes (e.g. homicidal attempts) or premeditated intoxications [4,5].

**Materials and methods:**

Tablets from benzodiazepine derivates (ATC codes: N05BA; national pharmacotherapeutic classification: 2.9.1. Anxiolytic, sedatives and hypnotics) were purchased from portuguese pharmacies. In the database were introduced the commercial name, the active substance name, the dose and also photographs were taken from the front, back and side and several physical characteristics of each pill were determined, namely: shape, surface elevation, logo and imprint description, break line, colour, coating, diameter, thickness, weight, horizontal, vertical and side view. The ISTI core is an identification algorithm targeted to matching results by score. The application is being prepared in a way that can either be accessed from a computer or from a mobile device. If economically viable, an image search and request by the users algorithm will be included, so that a photo can be an input, in a query which may prove particularly important to field in the future work. Furthermore, the system is being designed to be easily maintained, through a simple backoffice, and if necessary, agreements will be proposed to national regulatory authorities in order to assure that its data is effectively up to date.

**Results:**

The prototype developed application allows us to identify pills that may possibly be lost or out of their original packaging. This identification is made through a wide range of tablet features. The person who wants to identify a pill can access the ISTI and through answers to simple questions can find correspondence with the concerning tablet. **Discussion and conclusions:** The purpose of ISTI is to help health technicians, like doctors, nurses and pharmacists, and agents of authority to identify medicines pills which are not inside the original packaging. Although in the USA there are several Apps with the same objective, to the best of the authors knowledge, ISTI is the first App with this purpose in Portugal.
